# Subcutaneous daratumumab in Asian patients with heavily pretreated multiple myeloma: subgroup analyses of the noninferiority, phase 3 COLUMBA study

**DOI:** 10.1007/s00277-021-04405-2

**Published:** 2021-02-18

**Authors:** Shinsuke Iida, Takayuki Ishikawa, Chang Ki Min, Kihyun Kim, Su Peng Yeh, Saad Z. Usmani, Maria-Victoria Mateos, Hareth Nahi, Christoph Heuck, Xiang Qin, Dolly A. Parasrampuria, Katharine S. Gries, Ming Qi, Nizar Bahlis, Shigeki Ito

**Affiliations:** 1 Department of Hematology and Oncology, Nagoya City University Graduate School of Medical Sciences, 1 Kawasumi, Mizuho-cho, Mizuho-ku, Nagoya, 467-8601 Japan; 2 Department of Hematology, Kobe City Medical Center General Hospital, Kobe, Japan; 3 Seoul St. Mary’s Hospital, Seoul, South Korea; 4 Department of Medicine, Samsung Medical Center, Sungkyunkwan University School of Medicine, Seoul, South Korea; 5 China Medical University Hospital, Taichung, Taiwan; 6 Levine Cancer Institute/Atrium Health, Charlotte, NC USA; 7 University Hospital of Salamanca/IBSAL, Salamanca, Spain; 8 Karolinska Institute, Department of Medicine, Division of Hematology, Karolinska University Hospital at Huddinge, Stockholm, Sweden; 9 Janssen Research & Development, LLC, Spring House, PA USA; 10 Janssen Research & Development, LLC, Raritan, NJ USA; 11 Arnie Charbonneau Cancer Institute, University of Calgary, Calgary, Alberta Canada; 12 Division of Hematology and Oncology, Department of Internal Medicine, Iwate Medical University School of Medicine, Morioka, Iwate Japan

**Keywords:** Daratumumab, Multiple myeloma, Asian, Japanese, Monoclonal antibody, Subcutaneous

## Abstract

The phase 3 COLUMBA study demonstrated noninferiority of subcutaneous daratumumab (DARA SC) to intravenous daratumumab (DARA IV) in relapsed or refractory multiple myeloma. We present a subgroup analysis of Asian patients from COLUMBA. Eligible patients had ≥ 3 prior lines of therapy, including a proteasome inhibitor and an immunomodulatory drug, or were double refractory. Co-primary endpoints were overall response rate (ORR) and maximum trough concentration (*C*_trough_). Secondary endpoints included rates of infusion-related reactions, progression-free survival, and patient-reported satisfaction with therapy. Sixty-seven Asian patients (DARA SC, *n* = 30; DARA IV, *n* = 37) were randomized, including 42 Japanese patients (DARA SC, *n* = 18; DARA IV, *n* = 24). Comparable ORRs for DARA SC versus DARA IV were seen in the Asian cohort (66.7% vs 43.2%) and Japanese-only cohort (61.1% vs 54.2%), including patients weighing ≤ 65 kg. Similarity of *C*_trough_ was seen in both Asian and Japanese-only cohorts; the ratio of the geometric mean of the *C*_trough_ concentrations for DARA SC/DARA IV was 143.96% (90% confidence interval (CI), 112.03–185.00%) and 148.02% (90% CI, 113.32–193.34%), respectively. The Asian cohort (both treatment groups) and Japanese-only cohort (DARA SC group) experienced higher rates of grade 3/4 cytopenias compared with the global COLUMBA population, occurring predominantly in patients of low bodyweight; no patients discontinued treatment due to cytopenias. The Cancer Therapy Satisfaction Questionnaire results generally favored DARA SC. In the Asian and Japanese-only cohorts, DARA SC was comparable to DARA IV. The efficacy, pharmacokinetic, safety, and satisfaction results were generally consistent with the global COLUMBA population regardless of patient bodyweight. ClinicalTrials.gov Identifier: NCT03277105

**Supplementary Information:**

The online version contains supplementary material available at 10.1007/s00277-021-04405-2.

## Introduction

Daratumumab is a human IgGκ monoclonal antibody targeting CD38 with a direct on-tumor [1–4] and immunomodulatory [5–7] mechanism of action. Based on the positive efficacy and safety results from phase 1/2 (the GEN501 and SIRIUS studies) [8, 9] and phase 3 (the CASTOR, POLLUX, ALCYONE, MAIA, and CASSIOPEIA studies) [10–14] clinical trials, intravenous daratumumab (DARA IV) 16 mg/kg is approved in many countries as monotherapy and in combination with standard-of-care regimens for newly diagnosed multiple myeloma (NDMM) and relapsed or refractory multiple myeloma (RRMM) [15, 16].

DARA IV has median infusion durations during first, second, and subsequent infusions of 7, 4, and 3 hours, respectively [15]. To reduce the duration of administration of daratumumab, a subcutaneous version of daratumumab (DARA SC) co-formulated with recombinant human hyaluronidase PH20 (rHuPH20; ENHANZE^®^ drug delivery technology, Halozyme, Inc., San Diego, CA, USA) was developed that allows for dosing in 3–5 min [17, 18]. Flat dosing of DARA SC was selected based on population pharmacokinetics (PK) analysis and simulations for daratumumab and published literature that support the use of flat dosing for monoclonal antibodies [18–22]. The phase 1b PAVO study demonstrated that DARA SC 1800-mg flat dose had efficacy and PK comparable with DARA IV and lower rates of infusion-related reactions (IRRs) [17].

COLUMBA is a randomized, open-label, noninferiority, phase 3 study that evaluated the efficacy, PK, and safety of DARA SC versus DARA IV monotherapy in patients with heavily pretreated RRMM [23]. In the primary analysis of COLUMBA at a median follow-up of 7.5 months, DARA SC was noninferior to DARA IV in terms of the predefined noninferiority criteria evaluating the co-primary endpoints of overall response rate (ORR) and DARA maximum trough concentration (*C*_trough_; pre-dose on cycle 3 day 1) [23]. DARA SC also demonstrated a similar safety profile compared to DARA IV while reducing the median time of treatment administration and the rate of IRRs (12.7% vs 34.5%; *P* < 0.0001) [23]. With a longer median follow-up (13.7 months), DARA SC maintained noninferiority to DARA IV, and DARA SC continued to have a similar safety profile as DARA IV and shorter median administration duration and a reduction in IRR rates [24].

In Asian (Japanese, Korean, and Taiwanese) patients in the phase 3 POLLUX study of daratumumab in combination with lenalidomide and dexamethasone (D-Rd) or lenalidomide and dexamethasone (Rd) alone in patients with RRMM, D-Rd prolonged progression-free survival (PFS) and increased response rates versus Rd alone, consistent with the findings in the global POLLUX study population [25]. Similarly, consistent efficacy with the full study population was also seen in Asian (Japanese and Korean) patients in the phase 3 ALCYONE study of daratumumab in combination with bortezomib, melphalan, and prednisone (D-VMP) or bortezomib, melphalan, and prednisone (VMP) alone in patients with NDMM who were ineligible for autologous stem cell transplant (ASCT) compared to the results of the global ALCYONE study population [26]. In both studies, no new safety concerns were observed for Asian patients [25, 26].

To characterize DARA SC versus DARA IV in Asian patients with heavily pretreated RRMM, we performed a post hoc analysis of Asian (Japanese, Korean, and Taiwanese) patients enrolled in COLUMBA.

## Patients and methods

### Patients

A total of 67 Asian patients from the phase 3, randomized, open-label, multicenter, noninferiority COLUMBA study (ClinicalTrials.gov Identifier: NCT03277105) were included in this analysis. A separate subanalysis of the 42 Japanese patients alone was also conducted. An independent ethics committee or institutional review board approved the trial, and all patients provided written informed consent. The study protocol was conducted in accordance with the principles of the Declaration of Helsinki and the International Conference on Harmonisation guidelines on Good Clinical Practice.

The complete eligibility criteria have been described previously [23]. Briefly, eligible patients were ≥ 18 years of age, had documented RRMM with measurable disease at screening according to International Myeloma Working Group criteria [27, 28], received ≥ 3 prior lines of therapy (including a proteasome inhibitor (PI) and an immunomodulatory drug (IMiD)) or were refractory to both a PI and an IMiD, achieved at least a partial response (PR) to ≥ 1 prior line of therapy, and had an Eastern Cooperative Oncology Group performance status score of ≤ 2.

### Dosing

Patients were randomly assigned (1:1) according to planned stratification factors (bodyweight at baseline (≤ 65 kg, 66–85 kg, > 85 kg), number of prior lines of therapy (≤ 4 prior lines, > 4 prior lines), and type of myeloma (IgG, non-IgG)) [23]. Patients received DARA SC (1800-mg flat dose in a 15-mL solution administered by manual push over 3–5 min at left or right abdominal sites, alternating between individual doses) or DARA IV (16 mg/kg) given in 28-day cycles weekly for cycles 1 and 2, every 2 weeks for cycles 3–6, and every 4 weeks thereafter until disease progression.

### Evaluation and statistical analyses

The intent-to-treat (ITT) population included all patients randomized into the study. The PK-evaluable population included patients who received all 8 weekly doses of DARA IV or DARA SC in cycles 1 and 2 at the protocol-defined time points and provided a pre-dose PK sample on cycle 3 day 1. The safety population included all randomized patients who received ≥ 1 dose of daratumumab. The immunogenicity-evaluable population included patients who received ≥ 1 dose of daratumumab and had ≥ 1 serum sample after the start of the first dose of daratumumab.

The co-primary endpoints were ORR, defined as the number and proportion of patients achieving PR or better, and daratumumab maximum *C*_trough_ (pre-dose on cycle 3 day 1). Secondary endpoints included PFS, rates of IRRs, rate of very good partial response (VGPR) or better, rate of complete response (CR) or better, duration of and time to response, and patient-reported satisfaction with therapy. Patient-reported satisfaction with therapy was measured using a modified version of the Cancer Therapy Satisfaction Questionnaire (CTSQ), which included 9 items specific to satisfaction with therapy and a mean domain score that was calculated based on responses to 7 of 9 items [29]. Statistical analyses and additional methods used in COLUMBA were described previously [23] and are described briefly in the Supplementary information.

Exploratory subgroup analyses were conducted in the Asian and Japanese-only cohorts to assess the efficacy, safety, and PK of DARA SC versus DARA IV based on baseline bodyweight.

## Results

A total of 522 patients were randomized in COLUMBA; 67 (12.8%) patients were Asian (DARA SC, *n* = 30; DARA IV, *n* = 37), including 11 Korean (DARA SC, *n* = 4; DARA IV, *n* = 7), 14 Taiwanese (DARA SC, *n* = 8; DARA IV, *n* = 6), and 42 Japanese (DARA SC, *n* = 18; DARA IV, *n* = 24) patients. Baseline patient demographics, treatment history, and clinical characteristics are summarized in Table 1 and Supplementary Table 1 in the Supplementary information. In the Asian cohort, the median age was 70.0 (range, 33–84) years and the median bodyweight was 57.1 (range, 32.8–93.0) kg. In the Japanese-only cohort, the median age was 70.5 (range, 33–84) years and the median bodyweight was 52.9 (range, 32.8–83.2) kg. Only 1 patient in the Asian cohort (see Supplementary information for patient narrative) and no patients in the Japanese-only cohort had a bodyweight of > 85 kg. The Asian cohort received a median of 3.0 (range, 1–15) prior lines of therapy, and the Japanese-only cohort received a median of 4.0 (range, 1–15) prior lines of therapy. In the Asian cohort, 77.6% of patients were refractory to their last line of prior therapy, and 46.3% were refractory to both a PI and an IMiD. In the Japanese-only cohort, 69.0% of patients were refractory to the last line of prior therapy, and 54.8% were refractory to both a PI and an IMiD. A higher proportion of patients in the DARA SC group had high cytogenetic risk at baseline versus the DARA IV group (Asian, 30.8% vs 21.6%; Japanese-only, 37.5% vs 16.7%, respectively). A higher proportion of patients in the DARA SC group also had baseline grade ≥ 2 anemia and neutropenia (anemia: Asian, 46.7% vs 40.5%; Japanese-only, 55.6% vs 33.3%; neutropenia: Asian, 23.3% vs 16.2%; Japanese-only, 22.2% vs 8.3%, respectively).

**Table 1:** Demographic and baseline disease characteristics

	COLUMBA ITT population^a^		Asian	
	DARA IV (*n* = 259)	DARA SC (*n* = 263)	DARA IV (*n* = 37)	DARA SC (*n* = 30)
Age				
Median (range), years	68.0 (33–92)	65.0 (42–84)	70.0 (33–83)	70.5 (48–84)
18–< 65 years, *n* (%)	100 (38.6)	121 (46.0)	12 (32.4)	10 (33.3)
65–< 75 years, *n* (%)	100 (38.6)	95 (36.1)	18 (48.6)	9 (30.0)
≥ 75 years, *n* (%)	59 (22.8)	47 (17.9)	7 (18.9)	11 (36.7)
Male, *n* (%)	149 (57.5)	136 (51.7)	20 (54.1)	15 (50.0)
Bodyweight, kg				
*n*	258	262	37	30
Median (range)	73.0 (28.6–138.0)	72.4 (39.0–130.0)	56.7 (32.8–93.0)	60.1 (40.5–83.2)
≤ 65 kg, *n* (%)	92 (35.7)	94 (35.9)	31 (83.8)	24 (80.0)
ECOG PS score, *n* (%)				
0	88 (34.0)	64 (24.3)	23 (62.2)	14 (46.7)
1	132 (51.0)	152 (57.8)	12 (32.4)	14 (46.7)
2	38 (14.7)	47 (17.9)	2 (5.4)	2 (6.7)
> 2	1 (0.4)^b^	0	0	0
ISS disease stage,^c^ *n* (%)				
*n*	259	262	37	30
I	94 (36.3)	82 (31.3)	20 (54.1)	14 (46.7)
II	89 (34.4)	101 (38.5)	10 (27.0)	12 (40.0)
III	76 (29.3)	79 (30.2)	7 (18.9)	4 (13.3)
Type of myeloma, *n* (%)				
IgG	144 (55.6)	156 (59.3)	20 (54.1)	19 (63.3)
IgA	45 (17.4)	45 (17.1)	3 (8.1)	5 (16.7)
IgM	0	2 (0.8)	0	0
IgD	2 (0.8)	4 (1.5)	1 (2.7)	1 (3.3)
IgE	0	0	0	0
Light chain	62 (23.9)	53 (20.2)	12 (32.4)	5 (16.7)
Kappa	45 (17.4)	27 (10.3)	6 (16.2)	2 (6.7)
Lambda	15 (5.8)	23 (8.7)	6 (16.2)	2 (6.7)
Biclonal	6 (2.3)	3 (1.1)	1 (2.7)	0
Prior ASCT, *n* (%)	121 (46.7)	144 (54.8)	14 (37.8)	13 (43.3)
Prior lines of therapy, median (range)	4.0 (1–15)	4.0 (2–12)	3.0 (1–15)	3.5 (2–12)
Refractory to, *n* (%)				
Last prior line of therapy	220 (84.9)	209 (79.5)	31 (83.8)	21 (70.0)
PI and IMiD	133 (51.4)	125 (47.5)	21 (56.8)	10 (33.3)
Cytogenetic risk profile^d^				
*n*	202	198	37	26
Standard risk, *n* (%)	167 (82.7)	146 (73.7)	29 (78.4)	18 (69.2)
High risk, *n* (%)	35 (17.3)	52 (26.3)	8 (21.6)	8 (30.8)
*t*(4;14)	15 (7.4)	22 (11.1)	6 (16.2)	5 (19.2)
*t*(14;16)	4 (2.0)	7 (3.5)	1 (2.7)	2 (7.7)
del17p	22 (10.9)	32 (16.2)	5 (13.5)	3 (11.5)

*ITT*, intent-to-treat; *DARA*, daratumumab; *IV*, intravenous; *SC*, subcutaneous; *ECOG PS*, Eastern Cooperative Oncology Group performance status; *ISS*, International Staging System; *ASCT*, autologous stem cell transplant; *PI*, proteasome inhibitor; *IMiD*, immunomodulatory drug

^a^ITT population, defined as all randomized patients

^b^One patient who met the eligibility criteria with an ECOG PS score of 1 at screening was assessed with an ECOG PS score of 3 at cycle 1 day 1 as the baseline

^c^Based on the combination of serum β2-microglobulin and albumin

^d^Based on fluorescence in situ hybridization/karyotype testing

In the Asian cohort, 14 (46.7%) patients in the DARA SC group and 23 (62.2%) patients in the DARA IV group had discontinued treatment. The most common reason for discontinuation of treatment was progressive disease (DARA SC, 11 (36.7%) patients; DARA IV, 19 (51.4%) patients). Other reasons for discontinuation of treatment in the Asian cohort were adverse event, patient decision, physician decision (each observed in 1 patient in each group), and death (1 patient in the DARA IV group). In the Japanese-only cohort, 9 (50.0%) patients in the DARA SC group and 13 (54.2%) patients in the DARA IV group had discontinued treatment, most frequently due to progressive disease (DARA SC, 7 (38.9%) patients; DARA IV, 12 (50.0%) patients). Other reasons for discontinuation of treatment in the Japanese-only cohort were death (1 patient in the DARA IV group) and patient decision and physician decision (each observed in 1 patient in the DARA SC group).

In the Asian cohort, the median duration of treatment was 6.5 months in the DARA SC group and 5.6 months in the DARA IV group, and the median number of treatment cycles completed was similar (8.0 vs 6.0, respectively). In the Japanese-only cohort, the median duration of treatment was similar between DARA SC and DARA IV (6.5 months vs 6.4 months, respectively), and a median of 8 treatment cycles was completed in both the DARA SC and DARA IV groups.

In the Asian cohort, the median relative dose intensity of daratumumab was 100% for DARA SC and 99.7% for DARA IV for all treatment cycles. In the Japanese-only cohort, the median relative dose intensity of DARA was 100% for DARA SC and 99.9% for DARA IV for all treatment cycles. Consistent with results from the overall study population, in the Asian and Japanese-only cohorts, the median duration of infusion was reduced with DARA SC versus DARA IV during the first (Asian, 4 min vs 418 min; Japanese-only, 4 min vs 421 min), second (Asian, 4 min vs 260 min; Japanese-only, 4 min vs 261 min), and all subsequent infusions (Asian, 4 min vs 208 min; Japanese-only, 3 min vs 210 min), respectively.

### Efficacy

In the Asian cohort, the ORR was 66.7% for DARA SC versus 43.2% for DARA IV (Table 2). When assessed based on baseline bodyweight, ORRs for DARA SC versus DARA IV were 50.0% versus 41.2% in the ≤ 55-kg subgroup, 66.7% versus 35.5% in the ≤ 65-kg subgroup, and 66.7% versus 100% in the > 65–85–kg subgroup. Consistent with the global COLUMBA ITT population, comparable ORRs for DARA SC versus DARA IV were seen (relative risk, 1.54; 95% confidence interval (CI), 0.99–2.46), including in the ≤ 65-kg subgroup (relative risk, 1.88; 95% CI, 1.09–3.35). Rates of CR or better and VGPR or better were improved with DARA SC versus DARA IV, including in the ≤ 65-kg subgroup (Table 2). The median duration of response was not reached (NR) for DARA SC versus 10.4 (95% CI, 8.31–not estimable (NE)) months for DARA IV; similar results were seen in the ≤ 55-kg and ≤ 65-kg subgroups (Table 2).

**Table 2:** Overall best confirmed responses and duration of response

	COLUMBA ITT population		Asian		Asian ≤ 55 kg		Asian ≤ 65 kg		Asian > 65–85 kg	
	DARA IV (*n* = 259)	DARA SC (*n* = 263)	DARA IV (*n* = 37)	DARA SC (*n* = 30)	DARA IV (*n* = 17)	DARA SC (*n* = 12)	DARA IV (*n* = 31)	DARA SC (n = 24)	DARA IV (*n* = 5)	DARA SC (*n* = 6)
ORR, *n* (%)	102 (39.4)	114 (43.3)	16 (43.2)	20 (66.7)	7 (41.2)	6 (50.0)	11 (35.5)	16 (66.7)	5 (100.0)	4 (66.7)
≥ CR	12 (4.6)	8 (3.0)	1 (2.7)	3 (10.0)	1 (5.9)	0	1 (3.2)	3 (12.5)	0	0
sCR	3 (1.2)	2 (0.8)	0	2 (6.7)	0	0	0	2 (8.3)	0	0
CR	9 (3.5)	6 (2.3)	1 (2.7)	1 (3.3)	1 (5.9)	0	1 (3.2)	1 (4.2)	0	0
≥ VGPR	54 (20.8)	57 (21.7)	9 (24.3)	14 (46.7)	5 (29.4)	4 (33.3)	7 (22.6)	12 (50.0)	2 (40.0)	2 (33.3)
VGPR	42 (16.2)	49 (18.6)	8 (21.6)	11 (36.7)	4 (23.5)	4 (33.3)	6 (19.4)	9 (37.5)	2 (40.0)	2 (33.3)
PR	48 (18.5)	57 (21.7)	7 (18.9)	6 (20.0)	2 (11.8)	2 (16.7)	4 (12.9)	4 (16.7)	3 (60.0)	2 (33.3)
MR, *n* (%)	26 (10.0)	22 (8.4)	4 (10.8)	1 (3.3)	2 (11.8)	0	4 (12.9)	0	0	1 (16.7)
SD, *n* (%)	93 (35.9)	101 (38.4)	12 (32.4)	7 (23.3)	6 (35.3)	5 (41.7)	11 (35.5)	6 (25.0)	0	1 (16.7)
PD, *n* (%)	27 (10.4)	19 (7.2)	5 (13.5)	2 (6.7)	2 (11.8)	1 (8.3)	5 (16.1)	2 (8.3)	0	0
Not evaluable, *n* (%)	11 (4.2)	7 (2.7)	0	0	0	0	0	0	0	0
Duration of response, months										
*n*	102	114	16	20	7	6	11	16	5	4
Median	10.64	11.17	10.41	NR	10.41	NR	10.41	NR	NR	10.12
95% CI	9.26–NE	9.23–NE	8.31–NE	7.39–NE	8.31–NE	1.87–NE	8.31–NE	7.39–NE	2.30–NE	2.20–NE

*ITT*, intent-to-treat; *DARA*, daratumumab; *IV*, intravenous; *SC*, subcutaneous; *ORR*, overall response rate; *CR*, complete response; *sCR*, stringent complete response; *VGPR*, very good partial response; *PR*, partial response; *MR*, minimal response; *SD*, stable disease; *PD*, progressive disease; *NR*, not reached; *CI*, confidence interval; *NE*, not estimable

In the Japanese-only cohort, the ORR was 61.1% for DARA SC versus 54.2% for DARA IV (Supplementary Table 2 in the Supplementary information). ORRs were 45.5% versus 50.0% in the ≤ 55-kg subgroup, 58.8% versus 42.1% in the ≤ 65-kg subgroup, and 100% versus 100% in the > 65–85–kg subgroup. As in the Asian cohort, comparable ORRs for DARA SC versus DARA IV were seen (relative risk, 1.13; 95% CI, 0.64–1.93), including in the ≤ 65-kg subgroup (relative risk, 1.40; 95% CI, 0.72–2.80). Rates of VGPR or better were improved with DARA SC versus DARA IV (Supplementary Table 2). The median durations of response were similar to those reported for the Asian cohort (Table 2 and Supplementary Table 2).

**Fig. 1:**
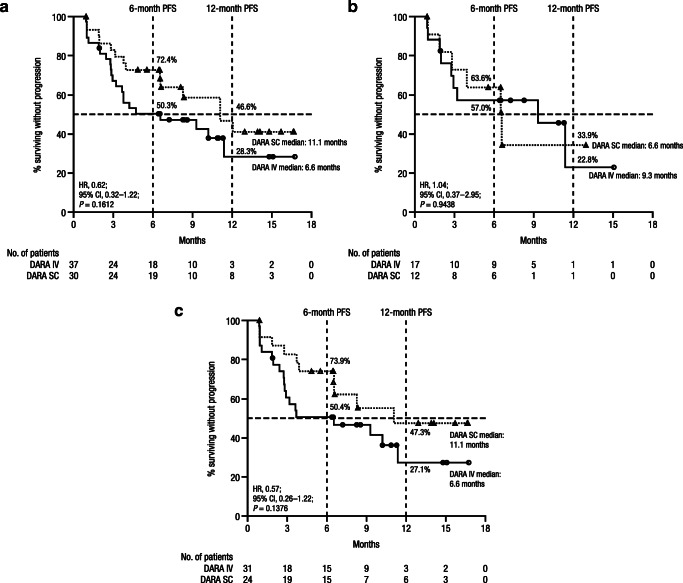
PFS of (**a**) Asian patients; (**b**) Asian patients with baseline bodyweight ≤ 55 kg; and (**c**) Asian patients with baseline bodyweight ≤ 65 kg. PFS, progression-free survival; DARA, daratumumab; SC, subcutaneous; IV, intravenous; HR, hazard ratio; CI, confidence interval

After a median follow-up of 13.7 (range, 0.03–19.4) months for the ITT population, the median PFS in the Asian cohort was 11.1 months with DARA SC versus 6.6 months with DARA IV (hazard ratio (HR), 0.62; 95% CI, 0.32–1.22; *P* = 0.1612; Fig. 1a); 6-month and 12-month PFS rates were 72.4% versus 50.3% and 46.6% versus 28.3%, respectively. In the ≤ 55-kg subgroup, the median PFS was 6.6 months with DARA SC versus 9.3 months with DARA IV (HR, 1.04; 95% CI, 0.37–2.95; *P* = 0.9438; Fig. 1b); 6-month and 12-month PFS rates were 63.6% versus 57.0% and 33.9% versus 22.8%, respectively. PFS results in the ≤ 65-kg subgroup were similar to those in the overall Asian cohort (Fig. 1c). In the > 65–85–kg subgroup, the median PFS was 11.1 months with DARA SC versus NR with DARA IV (HR, 1.01; 95% CI, 0.14–7.21; *P* = 0.9919); 6-month and 12-month PFS rates were 66.7% versus 60.0% and 44.4% versus NE, respectively.

In the Japanese-only cohort, the median PFS was 8.3 months with DARA SC versus 9.3 months with DARA IV (HR, 0.89; 95% CI, 0.36–2.16; *P* = 0.7870; Supplementary Fig. 1a in the Supplementary information); 6-month and 12-month PFS rates were 70.6% versus 54.2% and 34.3% versus 0%, respectively. In the ≤ 55-kg subgroup, the median PFS was 6.5 months with DARA SC versus 10.3 months with DARA IV (HR, 1.91; 95% CI, 0.53–6.86; *P* = 0.3122; Supplementary Fig. 1b in the Supplementary information). PFS results in the ≤ 65-kg subgroup were comparable to those in the overall Japanese-only cohort (Supplementary Fig. 1c in the Supplementary information). In the > 65–85–kg subgroup, the median PFS was NR with DARA SC or DARA IV (*P* = 0.5024); 6-month PFS rates were 100.0% versus 60.0%.

### Pharmacokinetics

**Fig. 2:**
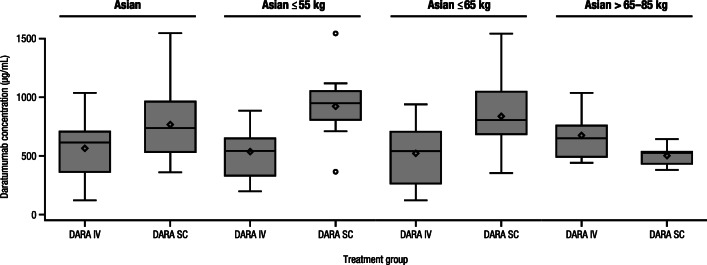
Maximum *C*_trough_ on cycle 3 day 1 of Asian patients by bodyweight subgroups. *C*_trough_, trough concentration; DARA, daratumumab; IV, intravenous; SC, subcutaneous. The boxes represent the 25th, 50th, and 75th percentiles, and the whiskers represent the furthest values from the median that did not exceed 1.5× interquartile range. Data above or below the respective whisker ends displayed as circles are considered outliers. The diamonds inside each box represent the algorithm mean

The co-primary endpoint of median maximum *C*_trough_ at cycle 3 day 1 for the DARA SC and DARA IV groups in the Asian cohort was 729 (range, 352–1543) μg/mL and 621 (range, 120–1036) μg/mL, respectively (Fig. 2); the ratio of the geometric mean of the maximum *C*_trough_ concentrations for DARA SC/DARA IV was 143.96% (90% CI, 112.03–185.00%). Consistent with the global COLUMBA PK-evaluable population, a similarity of *C*_trough_ was seen for DARA SC versus DARA IV, with lower bounds of the 90% CI for the ratio of geometric means of *C*_trough_ ≥ 80%. The median maximum *C*_trough_ for the DARA SC and DARA IV groups was 941 (range, 360–1543) μg/mL and 540 (range, 197–883) μg/mL in the ≤ 55-kg subgroup, 803 (range, 352–1543) μg/mL and 540 (range, 120–938) μg/mL in the ≤ 65-kg subgroup, and 526 (range, 378–640) μg/mL and 648 (range, 438–1036) μg/mL in the > 65–85–kg subgroup, respectively (Fig. 2).

Similar to the Asian cohort, the median maximum *C*_trough_ at cycle 3 day 1 for the DARA SC and DARA IV groups in the Japanese-only cohort was 814 (range, 360–1543) μg/mL and 592 (range 260–1036) μg/mL, respectively (Supplementary Fig. 2 in the Supplementary information); the ratio of the geometric mean of the maximum *C*_trough_ concentrations for DARA SC/DARA IV was 148.02% (90% CI, 113.32–193.34%), again demonstrating a similarity of *C*_trough_ for DARA SC versus DARA IV. The median maximum *C*_trough_ for the DARA SC and DARA IV groups was 887 (range, 360–1543) μg/mL and 540 (range, 328–883) μg/mL in the ≤ 55-kg subgroup, and 814 (range, 360–1543) μg/mL and 540 (range, 260–883) μg/mL in the ≤ 65-kg subgroup, respectively (Supplementary Fig. 2). The median maximum *C*_trough_ for the 5 PK-evaluable patients in the > 65–85–kg subgroup receiving DARA IV was 648 (range, 438–1036) μg/mL (Supplementary Fig. 2); no patients in this subgroup who received DARA SC were PK evaluable.

### Safety

The most common (> 25%) any-grade treatment-emergent adverse events (TEAEs) are summarized in Supplementary Table 3 in the Supplementary information. Although neutropenia was the most common hematologic TEAE in the Asian and Japanese-only cohorts, no treatment discontinuations or deaths occurred due to neutropenia. Febrile neutropenia was only reported with DARA IV in 2 non-Japanese Asian patients in the ≤ 65-kg subgroup.

In the Asian cohort, grade 3/4 TEAEs were reported in 16 (53.3%) patients in the DARA SC group and 21 (56.8%) patients in the DARA IV group (Supplementary Table 4 in the Supplementary information). Patients with baseline bodyweight ≤ 55 kg and ≤ 65 kg had higher rates of grade 3/4 TEAEs compared to those with baseline bodyweight > 65–85 kg (Supplementary Table 4). The most common (> 5%) grade 3/4 TEAEs are summarized in Table 3. The rates of grade 3/4 neutropenia (26.7% for DARA SC and 13.5% for DARA IV, respectively), lymphopenia (13.3% and 8.1%), and leukopenia (6.7% and 2.7%) were higher in the Asian cohort compared to the global COLUMBA safety population, whereas the rates of grade 3/4 anemia were similar (13.3% and 10.8%). Similarly, rates of grade 3/4 neutropenia, lymphopenia, and leukopenia were higher in the ≤ 55-kg and ≤ 65-kg subgroups compared to the global COLUMBA safety population. Rates of grade 3/4 neutropenia were also higher in the > 65–85–kg subgroup compared to the global COLUMBA safety population, whereas no patients in this subgroup experienced grade 3/4 lymphopenia or leukopenia.

**Table 3:** Most common (> 5%) grade 3/4 TEAEs

	COLUMBA safety population		Asian		Asian ≤ 55 kg		Asian ≤ 65 kg		Asian > 65–85 kg	
	DARA IV (*n* = 258)	DARA SC (*n* = 260)	DARA IV (*n* = 37)	DARA SC (*n* = 30)	DARA IV (*n* = 17)	DARA SC (*n* = 12)	DARA IV (*n* = 31)	DARA SC (*n* = 24)	DARA IV (*n* = 5)	DARA SC (*n* = 6)
Hematologic, *n* (%)										
Anemia	38 (14.7)	36 (13.8)	4 (10.8)	4 (13.3)	1 (5.9)	2 (16.7)	4 (12.9)	4 (16.7)	0	0
Thrombocytopenia	35 (13.6)	36 (13.8)	6 (16.2)	1 (3.3)	3 (17.6)	0	5 (16.1)	1 (4.2)	0	0
Neutropenia	20 (7.8)	34 (13.1)	5 (13.5)	8 (26.7)	2 (11.8)	3 (25.0)	5 (16.1)	6 (25.0)	0	2 (33.3)
Febrile neutropenia	6 (2.3)	3 (1.2)	2 (5.4)	0	0	0	2 (6.5)	0	0	0
Lymphopenia	16 (6.2)	14 (5.4)	3 (8.1)	4 (13.3)	2 (11.8)	2 (16.7)	3 (9.7)	4 (16.7)	0	0
Leukopenia	2 (0.8)	10 (3.8)	1 (2.7)	2 (6.7)	1 (5.9)	0	1 (3.2)	2 (8.3)	0	0
Nonhematologic, *n* (%)										
Hypertension	15 (5.8)	11 (4.2)	3 (8.1)	1 (3.3)	1 (5.9)	1 (8.3)	2 (6.5)	1 (4.2)	0	0
Pneumonia	12 (4.7)	10 (3.8)	4 (10.8)	0	1 (5.9)	0	4 (12.9)	0	0	0
Back pain	7 (2.7)	5 (1.9)	2 (5.4)	0	2 (11.8)	0	2 (6.5)	0	0	0
Hypercalcemia	6 (2.3)	5 (1.9)	2 (5.4)	0	1 (5.9)	0	2 (6.5)	0	0	0
Hyperglycemia	5 (1.9)	1 (0.4)	1 (2.7)	0	1 (5.9)	0	1 (3.2)	0	0	0
Sepsis	4 (1.6)	4 (1.5)	1 (2.7)	1 (3.3)	1 (5.9)	1 (8.3)	1 (3.2)	1 (4.2)	0	0
Hypokalemia	4 (1.6)	2 (0.8)	2 (5.4)	0	0	0	2 (6.5)	0	0	0
Bone pain	3 (1.2)	5 (1.9)	1 (2.7)	0	1 (5.9)	0	1 (3.2)	0	0	0
Hyperuricemia	2 (0.8)	1 (0.4)	1 (2.7)	0	1 (5.9)	0	1 (3.2)	0	0	0
Inguinal hernia	2 (0.8)	0	2 (5.4)	0	0	0	2 (6.5)	0	0	0
Mastoiditis	1 (0.4)	0	1 (2.7)	0	1 (5.9)	0	1 (3.2)	0	0	0
Neurosensory deafness	1 (0.4)	0	1 (2.7)	0	1 (5.9)	0	1 (3.2)	0	0	0
Ileus	1 (0.4)	0	1 (2.7)	0	1 (5.9)	0	1 (3.2)	0	0	0
Cancer pain	0	1 (0.4)	0	1 (3.3)	0	1 (8.3)	0	1 (4.2)	0	0
Pathologic fracture	0	1 (0.4)	0	1 (3.3)	0	0	0	0	0	1 (16.7)
Syncope	0	1 (0.4)	0	1 (3.3)	0	0	0	0	0	1 (16.7)

*TEAE*, treatment-emergent adverse event; *DARA*, daratumumab; *IV*, intravenous; *SC*, subcutaneous

In the Japanese-only cohort, grade 3/4 TEAEs were reported in 10 (55.6%) patients in the DARA SC group and 10 (41.7%) patients in the DARA IV group (Supplementary Table 4). As in the Asian cohort, the Japanese-only cohort had high rates of grade 3/4 neutropenia (27.8% for DARA SC and 0% for DARA IV, respectively), lymphopenia (16.7% and 8.3%), and leukopenia (11.1% and 4.2%; Supplementary Table 5 in the Supplementary information). Grade 3/4 anemia was reported at a higher rate with DARA SC (22.2%) compared to the global COLUMBA safety population and occurred in no patients receiving DARA IV. No patients in the > 65–85–kg subgroup experienced a grade 3/4 TEAE.

In the Asian cohort, any-grade infections were reported at similar rates for DARA SC and DARA IV (66.7% vs 67.6%, respectively), and a lower rate of grade 3/4 infections was observed with DARA SC versus DARA IV (3.3% vs 16.2%; Supplementary Table 4). A similar pattern was observed in the ≤ 55-kg and ≤ 65-kg subgroups (Supplementary Table 4). The higher rates of grade 3/4 neutropenia observed with DARA SC versus DARA IV across bodyweight subgroups did not result in higher rates of grade 3/4 infections (Table 3 and Supplementary Table 4). Medication for infection prophylaxis was used in 60% of patients in the DARA SC group and 51.4% of patients in the DARA IV group, with the highest rates of use among patients in the ≤ 55-kg subgroup receiving DARA SC (75.0%; Supplementary Table 4). The most common (> 2 patients in either treatment group) medications for infection prophylaxis were sulfamethoxazole/trimethoprim (33.3% and 21.6% of patients in the DARA SC and DARA IV groups, respectively) and acyclovir (50.0% and 35.1%). No patients receiving DARA SC died as a result of an infection versus 2 (5.4%) patients in the ≤ 65-kg subgroup receiving DARA IV (including 1 patient in the ≤ 55-kg subgroup). Neutropenic sepsis was not reported in any patients.

In the Japanese-only cohort, any-grade and grade 3/4 infections were reported at similar rates for DARA SC versus DARA IV (any grade, 61.1% vs 58.3%; grade 3/4, 5.6% vs 8.3%, respectively; Supplementary Table 4). Rates of any-grade infections were also reported at similar rates for DARA SC versus DARA IV for the ≤ 55-kg and ≤ 65-kg subgroups. Compared with the overall Japanese-only cohort, rates of grade 3/4 infections were higher in the ≤ 55-kg subgroup, while similar rates were observed in the ≤ 65-kg subgroup (Supplementary Table 4). Medication for infection prophylaxis was used in 83.3% of patients in the DARA SC group and 54.2% of patients in the DARA IV group; these medications were used most frequently in the ≤ 55-kg and ≤ 65-kg subgroups (Supplementary Table 4). Consistent with the Asian cohort, the most common (> 2 patients in either treatment group) medications for infection prophylaxis were sulfamethoxazole/trimethoprim (50.0% and 25.0% of patients in the DARA SC and DARA IV groups, respectively) and acyclovir (66.7% and 29.2%). No patients in the DARA SC group died as a result of an infection versus 1 (4.2%) patient in the ≤ 65-kg subgroup receiving DARA IV.

TEAEs leading to treatment discontinuations in the Asian cohort occurred in 1 (3.3%) patient with DARA SC (increased alanine and aspartate aminotransferase) and 1 (2.7%) patient with DARA IV (hepatitis B reactivation); all were in the ≤ 65-kg subgroup. No patients in the Japanese-only cohort discontinued treatment due to TEAEs. TEAEs leading to death in the Asian cohort occurred in 1 (3.3%) patient with DARA SC (general physical health deterioration in a Japanese patient in the ≤ 65-kg subgroup) and 2 (5.4%) patients with DARA IV (hepatitis B reactivation in 1 patient in the ≤ 65-kg subgroup and sepsis in 1 Japanese patient in the ≤ 55-kg subgroup).

In the Asian cohort, serious adverse events (SAEs) occurred in 4 (13.3%) patients in the DARA SC group and 15 (40.5%) patients in the DARA IV group; 1 SAE (sepsis) in the DARA SC group and 3 SAEs (sepsis, hepatitis B reactivation, and pneumonia) in the DARA IV group were considered related to daratumumab (Supplementary Table 4). Lower SAE rates were also seen with DARA SC versus DARA IV in the ≤ 55-kg and ≤ 65-kg subgroups (Supplementary Table 4).

In the Japanese-only cohort, SAEs occurred in 2 (11.1%) patients in the DARA SC group and 7 (29.2%) patients in the DARA IV group; only 1 SAE (sepsis) in each group was considered related to daratumumab (Supplementary Table 4). All patients who reported an SAE in the Japanese-only cohort had a baseline bodyweight of ≤ 65 kg.

In the Asian cohort, the IRR rate was lower with DARA SC versus DARA IV (10.0% vs 18.9%, respectively; odds ratio, 0.48; 95% CI, 0.11–2.03; *P* = 0.3120; Supplementary Table 4), which was similar to that reported in the global COLUMBA safety population (12.7% vs 34.5%; odds ratio, 0.28; 95% CI, 0.18–0.44; *P* < 0.0001). The IRR rate was also lower with DARA SC versus DARA IV in the ≤ 55-kg subgroup (16.7% vs 23.5%, respectively) and ≤ 65-kg subgroup (12.5% vs 19.4%). In the > 65–85–kg subgroup, only 1 patient receiving DARA IV experienced an IRR. All IRRs were mild and occurred primarily during the first administration; no grade 3/4 IRRs were reported. Median time to onset of IRRs was 27.5 (range, 3.2–52.0) hours for DARA SC and 1.7 (range, 1.0–24.5) hours for DARA IV (Supplementary Table 4). In the ≤ 55-kg and ≤ 65-kg subgroups, median time to onset of IRRs with DARA SC was longer than and comparable to that in the overall Asian cohort, respectively (Supplementary Table 4).

In the Japanese-only cohort, the IRR rate was the same for patients receiving DARA SC and DARA IV (16.7% vs 16.7%, respectively; Supplementary Table 4). The IRR rate was lower with DARA SC versus DARA IV in the ≤ 55-kg subgroup (18.2% vs 25.0%, respectively) and was similar between DARA SC and DARA IV in the ≤ 65-kg subgroup (17.6% vs 15.8%). Median time to onset of IRRs was 27.5 (range, 3.2–52.0) hours for DARA SC and 1.5 (range, 1.0–24.5) hours for DARA IV (Supplementary Table 4). The 1 Asian patient in the > 65–85–kg subgroup who experienced an IRR with DARA IV was Japanese; the time to IRR onset was 1 hour.

### Immunogenicity

No immunogenicity-evaluable patients in the Asian cohort (DARA SC, *n* = 29; DARA IV, *n* = 34) were positive for anti-daratumumab antibodies. Two non-Japanese patients in the DARA SC group were positive for anti-rHuPH20 antibodies at baseline, and 1 non-Japanese patient was positive for anti-rHuPH20 antibodies during treatment; all were nonneutralizing.

### Patient-reported satisfaction

**Fig. 3:**
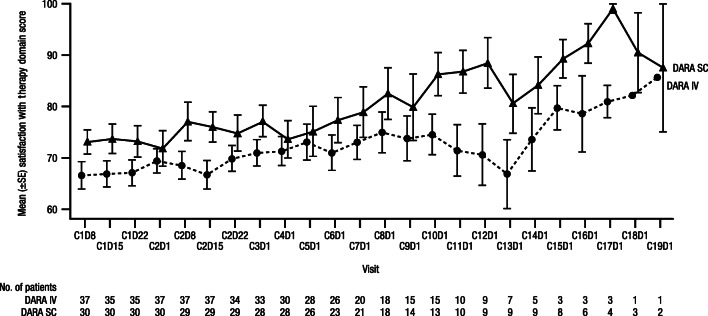
Mean modified CTSQ scores over time for the satisfaction with therapy domain in Asian patients. CTSQ, Cancer Therapy Satisfaction Questionnaire; SE, standard error; DARA, daratumumab; SC, subcutaneous; IV, intravenous; C, cycle; D, day

Overall, in the Asian cohort, patients receiving DARA SC were more satisfied with their cancer therapy than patients receiving DARA IV (Fig. 3). Compliance rates for the modified CTSQ assessments were high in the DARA SC and DARA IV groups (> 90% of patients) of both the Asian and Japanese-only cohorts. In the Asian cohort, the mean scores for the satisfaction with therapy domain were > 70 points (scale: 0–100; higher values indicate greater satisfaction) for DARA SC treatment and > 65 points for DARA IV treatment and were higher for DARA SC compared with DARA IV at all assessed time points. Additionally, more patients reported positive responses to DARA SC versus DARA IV treatment for the following 2 relevant components of the satisfaction with therapy domain that assessed the effect of the route of administration on patient satisfaction with therapy: “satisfied with form of cancer therapy” and “taking cancer therapy as difficult as expected” (Supplementary Figs. 4a and 5a in the Supplementary information). In the Japanese-only cohort, mean scores were similar between the DARA SC and DARA IV groups (Supplementary Figs. 3, 4b, and 5b in the Supplementary information).

## Discussion

In both the Asian and Japanese-only cohorts, comparable ORRs and maximum *C*_troughs_ were observed for DARA SC 1800-mg flat dose compared to DARA IV 16 mg/kg. Response rates and PFS were also similar between DARA SC and DARA IV, including in the lower-bodyweight subgroups. Overall, Asian patients receiving DARA SC were generally more satisfied with their cancer therapy compared to Asian patients receiving DARA IV. The results presented here for the Asian and Japanese-only cohorts overall and based on baseline bodyweight are generally consistent with those from the global COLUMBA study population [24]. At a median follow-up of 7.5 months (primary analysis), in the global population of the COLUMBA study, DARA SC was noninferior to DARA IV in terms of ORR (41.1% vs 37.1%, respectively; relative risk, 1.11; 95% CI, 0.89–1.37) and maximum *C*_trough_ (ratio of DARA SC/DARA IV, 107.93%; 90% CI, 95.74–121.67%) [23]. Median PFS was consistent between DARA SC and DARA IV (5.6 months vs 6.1 months, respectively; HR, 0.99; 95% CI, 0.78–1.26; *P* = 0.93). Additionally, DARA SC demonstrated a similar safety profile compared to DARA IV while reducing the median time of treatment administration and significantly reducing the rate of IRRs [23]. The noninferiority of DARA SC to DARA IV and similar median PFS and safety profile were also seen at a longer median follow-up of 13.7 months, while higher satisfaction with therapy was reported for DARA SC versus DARA IV [24].

Although grade 3/4 cytopenias occurred at higher rates in the Asian cohort for both DARA SC and DARA IV groups compared with the global COLUMBA safety population [24], this did not result in increased rates of severe infections and no patients discontinued treatment or died due to cytopenias, suggesting that they were well managed. Similarly, in the Japanese-only cohort, patients treated with DARA SC experienced higher rates of grade 3/4 cytopenias, but no Japanese patients discontinued treatment due to TEAEs. Grade 3/4 cytopenias in the Asian and Japanese-only cohorts occurred predominantly in patients of lower bodyweight. These results are generally consistent with the Asian and Japanese-only cohort subgroup analyses of ALCYONE, which showed higher rates of grade 3/4 cytopenias in the Asian and Japanese-only cohorts compared with the global ALCYONE safety population in both the D-VMP and VMP treatment groups [26].

The Asian cohort experienced a lower rate of IRRs with DARA SC versus DARA IV, consistent with observations for the global COLUMBA safety population [24]. The rate of IRRs was similar between the DARA SC group and the DARA IV group in the Japanese-only cohort. While the rate of IRRs with DARA SC was similar between the Asian (10.0%) and Japanese-only cohort (16.7%) and the global COLUMBA safety population (12.7%), the IRR rate was lower with DARA IV in the Asian (18.9%) and Japanese-only cohort (16.7%) compared to that of the global COLUMBA safety population (34.5%) and previous DARA IV studies in Asian (40–49.0%) and Japanese-only (35.0–62.5%) patients [25, 26, 30–32]. In a phase 1, dose-escalation DARA IV monotherapy study in Japanese patients, IRRs were reported in 4 of 9 (44.4%) patients (1 patient receiving DARA IV 8-mg/kg dose and 3 patients receiving DARA IV 16 mg/kg) [30].

Importantly, although the median baseline bodyweight in the Asian cohort was numerically lower and the median maximum daratumumab *C*_trough_ observed with DARA SC in the Asian cohort was numerically higher than in the global COLUMBA study population, the efficacy and safety of DARA SC among patients in the Asian cohort were generally consistent with those of the global COLUMBA study population [24]. Among non-Asian patients with baseline bodyweight ≤ 55 kg (DARA SC, *n* = 22; DARA IV, *n* = 23), the most common (> 15%) grade 3/4 TEAEs with DARA SC and DARA IV were neutropenia (22.7% and 0%, respectively), thrombocytopenia (18.2% and 8.7%), and anemia (9.1% and 26.1%). Among these patients, the rate of IRRs was lower with DARA SC versus DARA IV (18.2% vs 52.2%; odds ratio, 0.20; 95% CI, 0.05–0.79; *P* = 0.0185). In comparison, higher rates of grade 3/4 neutropenia and lymphopenia were reported with both DARA SC and DARA IV in the ≤ 55-kg Asian cohort compared with the ≤ 55-kg non-Asian cohort, but none led to treatment discontinuation. Although the small patient numbers limit comparisons, these differences in grade 3/4 cytopenia rates may indicate underlying differences between Asian and non-Asian patients of low bodyweight in terms of vulnerability to these events. The rate of IRRs was similarly lower with DARA SC versus DARA IV. These results are aligned with a subgroup analysis of the overall COLUMBA study population by patient bodyweight, which demonstrated that no dose individualization of DARA SC is necessary on the basis of bodyweight based on comparable efficacy and PK results across bodyweights [33].

In conclusion, DARA SC 1800-mg flat dose was comparable to DARA IV 16 mg/kg, with no new safety concerns, in Japanese, Korean, and Taiwanese patients. Although the analyses in this report are limited by small patient numbers, the efficacy and safety of DARA SC in Asian patients overall and of low bodyweight were consistent with those of the global COLUMBA population.

## Supplementary information

ESM 1 (PDF 981 kb)

## Data Availability

The data sharing policy of Janssen Pharmaceutical Companies of Johnson & Johnson is available at https://www.janssen.com/clinical-trials/transparency. As noted on this site, requests for access to the study data can be submitted through Yale Open Data Access (YODA) Project site at http://yoda.yale.edu.
