# Supplementary material for: Subcutaneous daratumumab in Asian patients with heavily pretreated multiple myeloma: subgroup analyses of the noninferiority, phase 3 COLUMBA study
Source: Ann Hematol. 2021 Feb 18;100(4):1065–77. doi: 10.1007/s00277-021-04405-2 (PMC7960612; doi:10.1007/s00277-021-04405-2)
Supplement: Supplementary file 1 — (PDF 981 kb) [file 277_2021_4405_MOESM1_ESM.pdf]

Electronic Supplementary Material

Subcutaneous daratumumab in Asian patients with heavily pretreated multiple myeloma: subgroup analyses of the noninferiority, phase 3 COLUMBA study

Annals of Hematology

Shinsuke Iida, Takayuki Ishikawa, Chang Ki Min, Kihyun Kim, Su Peng Yeh, Saad Z. Usmani, Maria-Victoria Mateos, Hareth Nahi, Christoph Heuck, Xiang Qin, Dolly A. Parasrampuria, Katharine S. Gries, Ming Qi, Nizar Bahlis, Shigeki Ito

Corresponding author:
Shinsuke Iida
Department of Hematology and Oncology
Nagoya City University Graduate School of Medical Sciences, Nagoya, Japan
1 Kawasumi, Mizuho-cho, Mizuho-ku Nagoya 467-8601, Japan
Phone: (052) 853-8736
Email: iida@med.nagoya-cu.ac.jp

Supplementary Methods

Evaluation and statistical analyses

For the ITT population, noninferiority of ORR for DARA SC versus DARA IV was defined using a 60% retention level from the SIRIUS study [1]. The noninferiority test for nonutility null was based on the Farrington–Manning test [2]. For ORR, noninferiority of DARA SC to DARA IV was established if the lower bound of the 95% CI was ≥60%, and superiority of DARA SC to DARA IV was to be concluded if noninferiority was established and the lower bound of the 95% CI of the relative risk was ≥100%. For maximum C_{trough}, noninferiority of DARA SC to DARA IV was determined if the lower bound of the 90% CI for the ratio of geometric means of maximum C_{trough} was ≥80%.

The Kaplan–Meier method was used to estimate PFS. The stratified log-rank test was used to compare PFS distributions between treatment groups, and a stratified Cox regression model with treatment as the sole explanatory variable was used to estimate HRs and 2-sided 95% CIs. The stratified Cochran–Mantel–Haenszel test was used to compare IRR rates and response rates between treatment groups, and odds ratios and 2-sided 95% CIs were provided. Duration of response, PK, safety, immunogenicity, and patient-reported outcomes were summarized using descriptive statistics.

Patient narrative

One Asian patient (non-Japanese) who received DARA IV had a baseline bodyweight of >85 kg. The patient had baseline grade 2 neutropenia. The patient achieved a best confirmed response of stable disease, and a maximum C_{trough} of 659 μg/mL. Any-grade TEAEs reported for the patient

were thrombocytopenia, upper abdominal pain, constipation, upper respiratory tract infection, hyperkalemia, hypomagnesemia, arthralgia, acute kidney injury, renal impairment, benign prostatic hyperplasia, cough, and hypertension. Grade 3/4 TEAEs reported for the patient were thrombocytopenia, hyperkalemia, acute kidney injury, renal impairment, and hypertension. The patient experienced an SAE of acute kidney injury that was not considered to be related to daratumumab. The patient did not experience an IRR. Medication for prophylaxis of infections used in the patient were ceftriaxone, benzydamine, acyclovir, and metronidazole. The patient did not discontinue treatment due to TEAEs.

References

1. Lonial S, Weiss BM, Usmani S, Singhal S, Chari A, Bahlis N, Belch A, Krishnan A, Vescio R, Mateos MV, Mazumder A, Orlowski RZ, Sutherland H, Blade J, Scott EC, Oriol A, Berdeja JG, Gharibo M, Stevens DA, LeBlanc R, Sebag M, Callander N, Jakubowiak A, White D, De La Rubia J, Richardson PG, Lisby S, Feng H, Uhlar CM, Khan I, Ahmadi T, Voorhees P (2016) Daratumumab monotherapy in patients with treatment-refractory multiple myeloma (SIRIUS): an open-label, randomised, phase 2 trial. Lancet 387:1551-1560

2. Farrington CP, Manning G (1990) Test statistics and sample size formulae for comparative binomial trials with null hypothesis of non-zero risk difference or non-unity relative risk. Stat Med 9:1447-1454

Supplementary Table 1 Demographic and Baseline Disease Characteristics of Japanese Patients

	Japanese-only	
	**DARA IV (n = 24)**	**DARA SC (n = 18)**
Age		
Median (range), years	70.5 (33-83)	70.5 (48-84)
18-<65 years, n (%)	6 (25.0)	6 (33.3)
65-<75 years, n (%)	13 (54.2)	5 (27.8)
≥75 years, n (%)	5 (20.8)	7 (38.9)
Male, n (%)	13 (54.2)	7 (38.9)
Bodyweight, kg		
Median (range)	54.1 (32.8-77.0)	52.6 (40.5-83.2)
≤65 kg, n (%)	19 (79.2)	17 (94.4)
ECOG PS score, n (%)		
0	18 (75.0)	13 (72.2)
1	5 (20.8)	4 (22.2)
2	1 (4.2)	1 (5.6)
>2	0	0
ISS disease stage,[a] n (%)		
I	16 (66.7)	8 (44.4)
II	5 (20.8)	6 (33.3)
III	3 (12.5)	4 (22.2)
Type of myeloma, n (%)		
IgG	13 (54.2)	12 (66.7)
IgA	1 (4.2)	2 (11.1)
IgM	0	0
IgD	0	0
IgE	0	0
Light chain	9 (37.5)	4 (22.2)
Kappa	5 (20.8)	2 (11.1)
Lambda	4 (16.7)	1 (5.6)
Biclonal	1 (4.2)	0
Prior ASCT, n (%)	10 (41.7)	9 (50.0)
Prior lines of therapy, median (range)	4.0 (1-15)	4.0 (2-12)
Refractory to, n (%)		
Last prior line of therapy	18 (75.0)	11 (61.1)
PI and IMiD	15 (62.5)	8 (44.4)
Cytogenetic risk profile[b]		
n	24	16
Standard risk, n (%)	20 (83.3)	10 (62.5)
High risk, n (%)	4 (16.7)	6 (37.5)
t(4;14)	3 (12.5)	4 (25.0)
t(14;16)	0	1 (6.3)
del17p	2 (8.3)	3 (18.8)

DARA, daratumumab; IV, intravenous; SC, subcutaneous; ECOG PS, Eastern Cooperative Oncology Group performance status; ISS, International Staging System; ASCT, autologous stem cell transplantation; PI, proteasome inhibitor; IMiD, immunomodulatory drug.

[a]Based on the combination of serum β2-microglobulin and albumin.

[b]Based on fluorescence in situ hybridization/karyotype testing.

Supplementary Table 2 Overall Best Confirmed Responses and Duration of Response in Japanese Patients by Bodyweight Subgroups

	Japanese-only		Japanese ≤55 kg		Japanese ≤65 kg		Japanese >65-85 kg	
	**DARA IV (n = 24)**	**DARA SC (n = 18)**	**DARA IV (n = 12)**	**DARA SC (n = 11)**	**DARA IV (n = 19)**	**DARA SC (n = 17)**	**DARA IV (n = 5)**	**DARA SC (n = 1)**
ORR, n (%)	13 (54.2)	11 (61.1)	6 (50.0)	5 (45.5)	8 (42.1)	10 (58.8)	5 (100.0)	1 (100.0)
≥CR	1 (4.2)	0	1 (8.3)	0	1 (5.3)	0	0	0
sCR	0	0	0	0	0	0	0	0
CR	1 (4.2)	0	1 (8.3)	0	1 (5.3)	0	0	0
≥VGPR	6 (25.0)	7 (38.9)	4 (33.3)	3 (27.3)	4 (21.1)	6 (35.3)	2 (40.0)	1 (100.0)
VGPR	5 (20.8)	7 (38.9)	3 (25.0)	3 (27.3)	3 (15.8)	6 (35.3)	2 (40.0)	1 (100.0)
PR	7 (29.2)	4 (22.2)	2 (16.7)	2 (18.2)	4 (21.1)	4 (23.5)	3 (60.0)	0
MR, n (%)	3 (12.5)	0	1 (8.3)	0	3 (15.8)	0	0	0
SD, n (%)	4 (16.7)	5 (27.8)	3 (25.0)	5 (45.5)	4 (21.1)	5 (29.4)	0	0
PD, n (%)	4 (16.7)	2 (11.1)	2 (16.7)	1 (9.1)	4 (21.1)	2 (11.8)	0	0
Not evaluable, n (%)	0	0	0	0	0	0	0	0
Duration of response, months								
n	13	11	6	5	8	10	5	1
Median	10.41	NR	10.41	NR	10.41	NR	NR	NR
95% CI	8.31-10.41	4.53-NE	8.31-10.41	1.87-NE	8.31-10.41	1.87-NE	2.30-NE	NE-NE

DARA, daratumumab; IV, intravenous; SC, subcutaneous; ORR, overall response rate; CR, complete response; sCR, stringent complete response; VGPR, very good partial response; PR, partial response; MR, minimal response; SD, stable disease; PD, progressive disease; NE, not estimable; NR, not reached; CI, confidence interval.

Supplementary Table 3 Most Common (>25%) Any-grade TEAEs

	**COLUMBA safety population**		**Asian**		**Asian ≤55 kg**		**Asian ≤65 kg**		**Asian >65-85 kg**		**Japanese-only**		**Japanese ≤55 kg**		**Japanese ≤65 kg**		**Japanese >65-85 kg**	
	**DARA IV (n = 258)**	**DARA SC (n = 260)**	**DARA IV (n = 37)**	**DARA SC (n = 30)**	**DARA IV (n =17)**	**DARA SC (n = 12)**	**DARA IV (n =31)**	**DARA SC (n = 24)**	**DARA IV (n = 5)**	**DARA SC (n = 6)**	**DARA IV (n = 24)**	**DARA SC (n = 18)**	**DARA IV (n = 12)**	**DARA SC (n = 11)**	**DARA IV (n = 19)**	**DARA SC (n = 17)**	**DARA IV (n = 5)**	**DARA SC (n = 1)**
Hematologic, n (%)																		
Anemia	64 (24.8)	71 (27.3)	4 (10.8)	6 (20.0)	1 (5.9)	3 (25.0)	4 (12.9)	6 (25.0)	0	0	0	5 (27.8)	0	3 (27.3)	0	5 (29.4)	0	0
Thrombocytopenia	49 (19.0)	51 (19.6)	10 (27.0)	3 (10.0)	5 (29.4)	0	8 (25.8)	3 (12.5)	1 (20.0)	0	4 (16.7)	1 (5.6)	2 (16.7)	0	3 (15.8)	1 (5.9)	1 (20.0)	0
Neutropenia	35 (13.6)	51 (19.6)	5 (13.5)	10 (33.3)	2 (11.8)	4 (33.3)	5 (16.1)	8 (33.3)	0	2 (33.3)	0	7 (38.9)	0	4 (36.4)	0	7 (41.2)	0	0
Lymphopenia	17 (6.6)	20 (7.7)	3 (8.1)	4 (13.3)	2 (11.8)	2 (16.7)	3 (9.7)	4 (16.7)	0	0	2 (8.3)	3 (16.7)	2 (16.7)	2 (18.2)	2 (10.5)	3 (17.6)	0	0
Nonhematologic, n (%)																		
Pyrexia	36 (14.0)	37 (14.2)	6 (16.2)	6 (20.0)	3 (17.6)	4 (33.3)	6 (19.4)	6 (25.0)	0	0	1 (4.2)	3 (16.7)	1 (8.3)	3 (27.3)	1 (5.3)	3 (17.6)	0	0
Upper respiratory tract infection	29 (11.2)	41 (15.8)	5 (13.5)	8 (26.7)	2 (11.8)	2 (16.7)	4 (12.9)	6 (25.0)	0	2 (33.3)	1 (4.2)	2 (11.1)	1 (8.3)	1 (9.1)	1 (5.3)	2 (11.8)	0	0
Nasopharyngitis	19 (7.4)	25 (9.6)	10 (27.0)	8 (26.7)	4 (23.5)	4 (33.3)	7 (22.6)	7 (29.2)	3 (60.0)	1 (16.7)	8 (33.3)	5 (27.8)	3 (25.0)	3 (27.3)	5 (26.3)	4 (23.5)	3 (60.0)	1 (100.0)
Arthralgia	18 (7.0)	28 (10.8)	2 (5.4)	3 (10.0)	0	1 (8.3)	0	1 (4.2)	1 (20.0)	2 (33.3)	1 (4.2)	1 (5.6)	0	1 (9.1)	0	1 (5.9)	1 (20.0)	0
Pain in extremity	12 (4.7)	18 (6.9)	3 (8.1)	3 (10.0)	2 (11.8)	1 (8.3)	3 (9.7)	1 (4.2)	0	2 (33.3)	1 (4.2)	1 (5.6)	1 (8.3)	1 (9.1)	1 (5.3)	1 (5.9)	0	0
Dizziness	11 (4.3)	13 (5.0)	3 (8.1)	5 (16.7)	1 (5.9)	1 (8.3)	3 (9.7)	3 (12.5)	0	2 (33.3)	1 (4.2)	1 (5.6)	1 (8.3)	1 (9.1)	1 (5.3)	1 (5.9)	0	0
Malaise	4 (1.6)	3 (1.2)	3 (8.1)	3 (10.0)	3 (17.6)	1 (8.3)	3 (9.7)	2 (8.3)	0	1 (16.7)	3 (12.5)	3 (16.7)	3 (25.0)	1 (9.1)	3 (15.8)	2 (11.8)	0	1 (100.0)
Increased C-reactive protein	3 (1.2)	4 (1.5)	0	2 (6.7)	0	1 (8.3)	0	1 (4.2)	0	1 (16.7)	0	2 (11.1)	0	1 (9.1)	0	1 (5.9)	0	1 (100.0)

TEAE, treatment-emergent adverse event; DARA, daratumumab; IV, intravenous; SC, subcutaneous.

Supplementary Table 4 Summary of Safety Results

	**COLUMBA Safety population**		**Asian**		**Asian ≤55 kg**		**Asian ≤65 kg**		**Asian >65-85 kg**		**Japanese-only**		**Japanese ≤55 kg**		**Japanese ≤65 kg**		**Japanese >65-85 kg**	
	**DARA IV (n = 258)**	**DARA SC (n = 260)**	**DARA IV (n = 37)**	**DARA SC (n = 30)**	**DARA IV (n =17)**	**DARA SC (n = 12)**	**DARA IV (n =31)**	**DARA SC (n = 24)**	**DARA IV (n = 5)**	**DARA SC (n = 6)**	**DARA IV (n = 24)**	**DARA SC (n = 18)**	**DARA IV (n = 12)**	**DARA SC (n = 11)**	**DARA IV (n = 19)**	**DARA SC (n = 17)**	**DARA IV (n = 5)**	**DARA SC (n = 1)**
Any-grade TEAE, n (%)	237 (91.9)	233 (89.6)	36 (97.3)	30 (100.0)	16 (94.1)	12 (100.0)	30 (96.8)	24 (100.0)	5 (100.0)	6 (100.0)	23 (95.8)	18 (100.0)	11 (91.7)	11 (100.0)	18 (94.7)	17 (100.0)	5 (100.0)	1 (100.0)
Any-grade infection	129 (50.0)	131 (50.4)	25 (67.6)	20 (66.7)	9 (52.9)	7 (58.3)	21 (67.7)	16 (66.7)	3 (60.0)	4 (66.7)	14 (58.3)	11 (61.1)	6 (50.0)	6 (54.5)	11 (57.9)	10 (58.8)	3 (60.0)	1 (100.0)
Grade 3/4 TEAE, n (%)	134 (51.9)	127 (48.8)	21 (56.8)	16 (53.3)	12 (70.6)	7 (58.3)	20 (64.5)	13 (54.2)	0	3 (50.0)	10 (41.7)	10 (55.6)	8 (66.7)	6 (54.5)	10 (52.6)	10 (58.8)	0	0
Grade 3/4 infection	37 (14.3)	32 (12.3)	6 (16.2)	1 (3.3)	3 (17.6)	1 (8.3)	6 (19.4)	1 (4.2)	0	0	2 (8.3)	1 (5.6)	2 (16.7)	1 (9.1)	2 (10.5)	1 (5.9)	0	0
Use of medications for infection prophylaxis, n (%)	62 (24.0)	65 (25.0)	19 (51.4)	18 (60.0)	8 (47.1)	9 (75.0)	17 (54.8)	15 (62.5)	1 (20.0)	3 (50.0)	13 (54.2)	15 (83.3)	7 (58.3)	9 (81.8)	12 (63.2)	14 (82.4)	1 (20.0)	1 (100.0)
SAE, n (%)	88 (34.1)	76 (29.2)	15 (40.5)	4 (13.3)	7 (41.2)	1 (8.3)	14 (45.2)	2 (8.3)	0	2 (33.3)	7 (29.2)	2 (11.1)	5 (41.7)	1 (9.1)	7 (36.8)	2 (11.8)	0	0
Related to daratumumab	22 (8.5)	18 (6.9)	3 (8.1)	1 (3.3)	1 (5.9)	1 (8.3)	3 (9.7)	1 (4.2)	0	0	1 (4.2)	1 (5.6)	1 (8.3)	1 (9.1)	1 (5.3)	1 (5.9)	0	0
Any-grade IRRs, n (%)	89 (34.5)	33 (12.7)	7 (18.9)	3 (10.0)	4 (23.5)	2 (16.7)	6 (19.4)	3 (12.5)	1 (20.0)	0	4 (16.7)	3 (16.7)	3 (25.0)	2 (18.2)	3 (15.8)	3 (17.6)	1 (20.0)	0
Time to onset of IRRs, hours																		
Median	1.5	3.6	1.7	27.5	1.5	47.8	1.8	27.5	1.0	–	1.5	27.5	1.5	47.8	1.5	27.5	1.0	–
Range	0-24.5	1.0-52.0	1.0-24.5	3.2-52.0	1.4-24.5	27.5-52.0	1.4-24.5	3.2-52.0	1.0-1.0	–	1.0-24.5	3.2-52.0	1.4-24.5	27.5-52.0	1.4-24.5	3.2-52.0	1.0-1.0	–

DARA, daratumumab; IV, intravenous; SC, subcutaneous; TEAE, treatment-emergent adverse event; SAE, serious adverse event; IRR, infusion-related reaction.

Supplementary Table 5 Most Common (>5%) Grade 3/4 TEAEs in Japanese Patients by Bodyweight Subgroups

	**Japanese-only**		**Japanese ≤55 kg**		**Japanese ≤65 kg**		**Japanese >65-85 kg**	
	**DARA IV (n = 24)**	**DARA SC (n = 18)**	**DARA IV (n = 12)**	**DARA SC (n = 11)**	**DARA IV (n = 19)**	**DARA SC (n = 17)**	**DARA IV (n = 5)**	**DARA SC (n = 1)**
Hematologic, n (%)								
Lymphopenia	2 (8.3)	3 (16.7)	2 (16.7)	2 (18.2)	2 (10.5)	3 (17.6)	0	0
Leukopenia	1 (4.2)	2 (11.1)	1 (8.3)	0	1 (5.3)	2 (11.8)	0	0
Neutropenia	0	5 (27.8)	0	3 (27.3)	0	5 (29.4)	0	0
Anemia	0	4 (22.2)	0	2 (18.2)	0	4 (23.5)	0	0
Thrombocytopenia	0	1 (5.6)	0	0	0	1 (5.9)	0	0
Nonhematologic, n (%)								
Inguinal hernia	2 (8.3)	0	0	0	2 (10.5)	0	0	0
Sepsis	1 (4.2)	1 (5.6)	1 (8.3)	1 (9.1)	1 (5.3)	1 (5.9)	0	0
Hypertension	1 (4.2)	0	1 (8.3)	0	1 (5.3)	0	0	0
Back pain	1 (4.2)	0	1 (8.3)	0	1 (5.3)	0	0	0
Bone pain	1 (4.2)	0	1 (8.3)	0	1 (5.3)	0	0	0
Hyperglycemia	1 (4.2)	0	1 (8.3)	0	1 (5.3)	0	0	0
Mastoiditis	1 (4.2)	0	1 (8.3)	0	1 (5.3)	0	0	0
Neurosensory deafness	1 (4.2)	0	1 (8.3)	0	1 (5.3)	0	0	0
Ileus	1 (4.2)	0	1 (8.3)	0	1 (5.3)	0	0	0
Cancer pain	0	1 (5.6)	0	1 (9.1)	0	1 (5.9)	0	0
General physical health deterioration	0	1 (5.6)	0	0	0	1 (5.9)	0	0
Cataract	0	1 (5.6)	0	0	0	1 (5.9)	0	0

TEAE, treatment-emergent adverse event; DARA, daratumumab; IV, intravenous; SC, subcutaneous.

Supplementary Fig. 1 PFS of (a) Japanese patients; (b) Japanese patients with baseline weight ≤55 kg; and (c) Japanese patients with baseline weight ≤65 kg

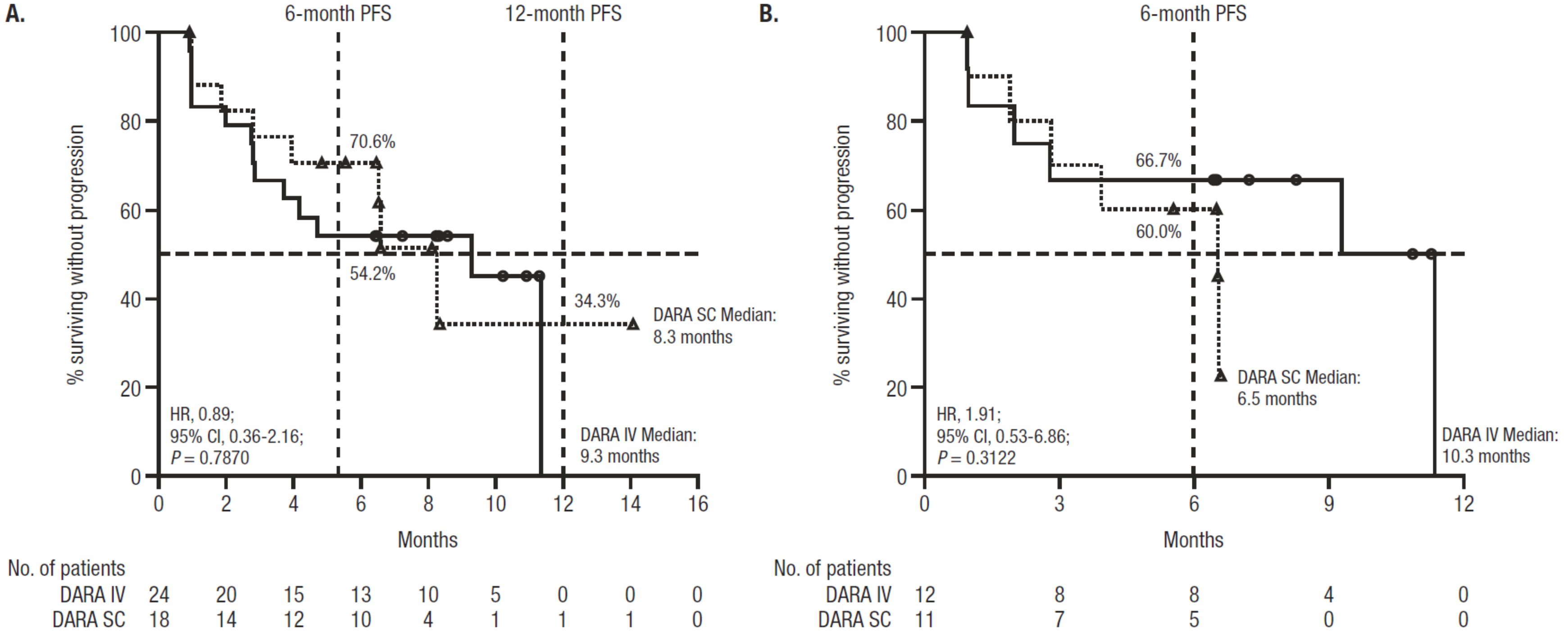

C.

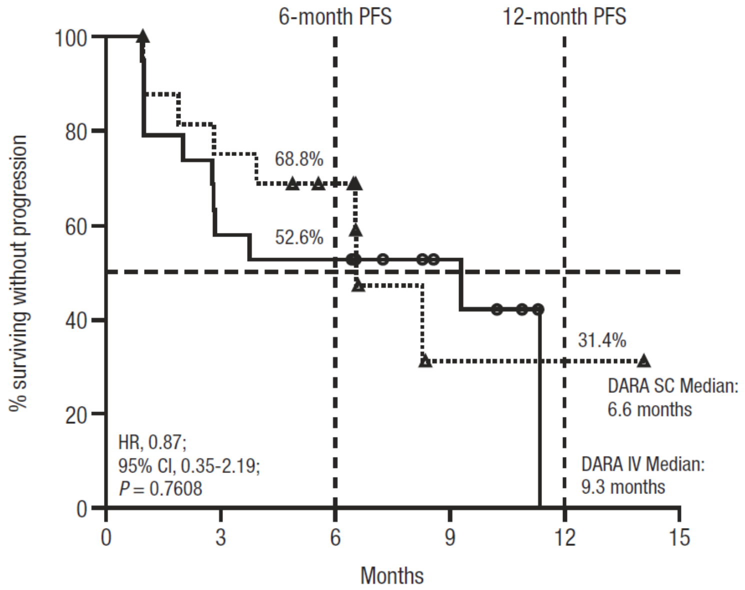

No. of patients						
DARA IV	19	11	10	5	0	0
DARA SC	17	12	9	1	1	0

PFS, progression-free survival; DARA, daratumumab; IV, intravenous; SC, subcutaneous; HR, hazard ratio; CI, confidence interval.

Supplementary Fig. 2 Maximum C_{trough} on Cycle 3 Day 1 of Japanese patients by bodyweight subgroups

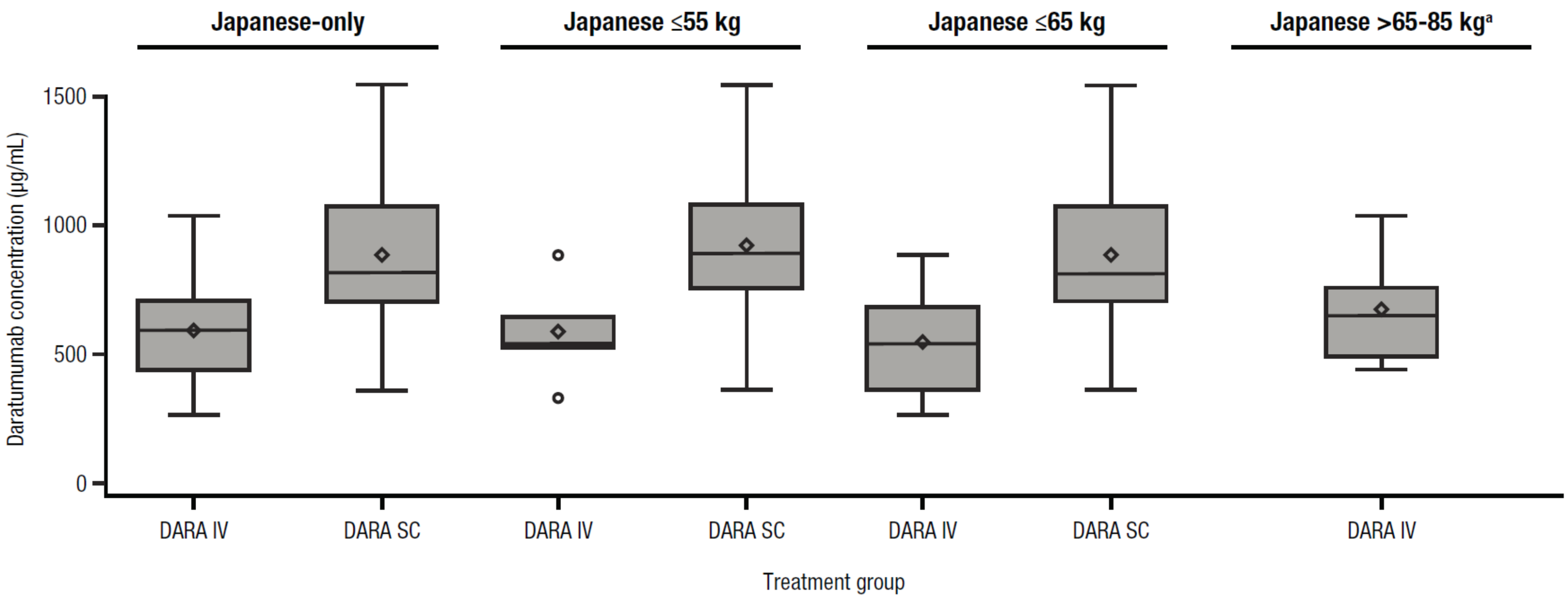

C_{trough}, trough concentration; DARA, daratumumab; IV, intravenous; SC, subcutaneous; PK, pharmacokinetic.
The boxes represent the 25th, 50th, and 75th percentiles, and the whiskers represent the furthest values from the median that did not exceed 1.5× interquartile range.
Data above or below the respective whisker ends displayed as circles are considered outliers. The diamonds inside each box represent the algorithm mean.
[a]No patients receiving DARA SC in this subgroup were PK evaluable.

Supplementary Fig. 3 Mean modified CTSQ scores over time for the Satisfaction With Therapy domain in Japanese patients

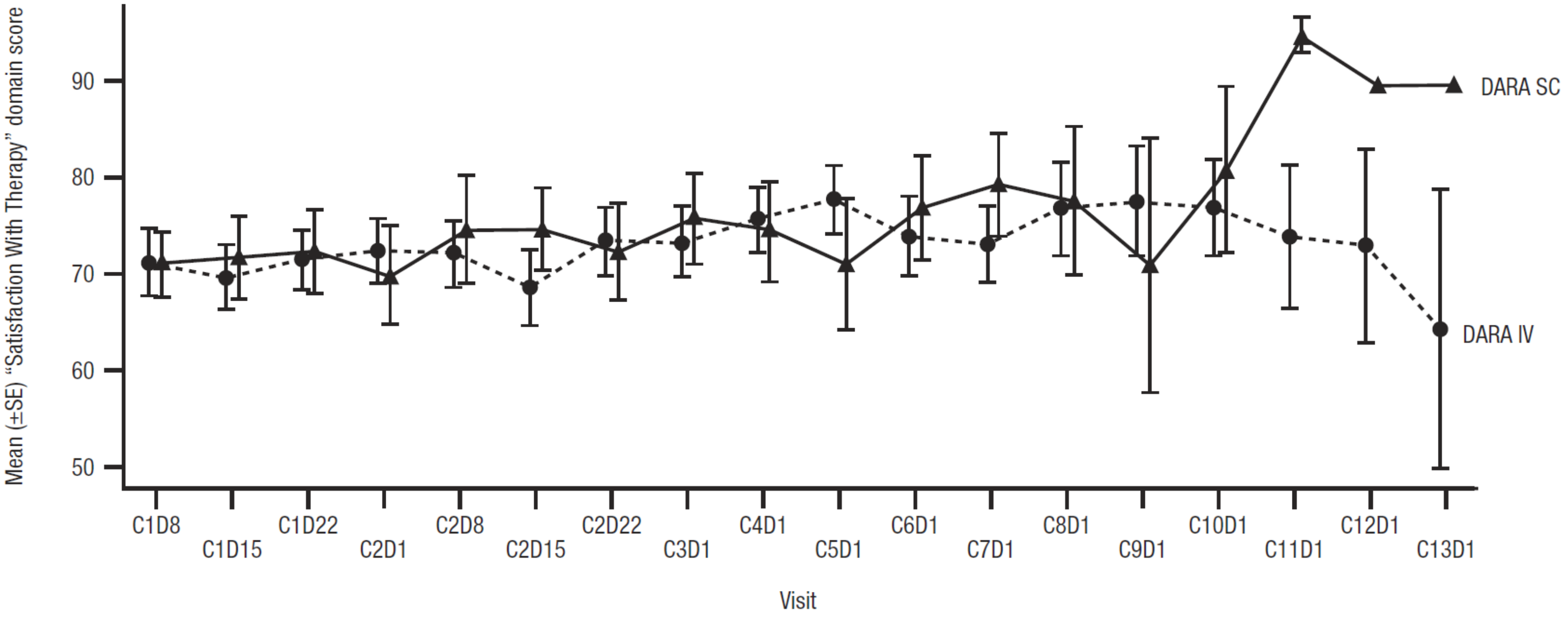

No. of patients																		
DARA IV	24	22	23	24	24	24	21	21	20	19	18	15	13	10	10	6	5	3
DARA SC	18	18	18	18	17	17	17	16	16	15	14	12	10	6	5	2	1	1

CTSQ, Cancer Therapy Satisfaction Questionnaire; SE, standard error; C, cycle; D, day; DARA, daratumumab; IV, intravenous; SC, subcutaneous.

Supplementary Fig. 4 Mean modified CTSQ scores over time for the "Satisfied With Form of Cancer Therapy" component of the Satisfaction With Therapy domain in (a) Asian and (b) Japanese patients

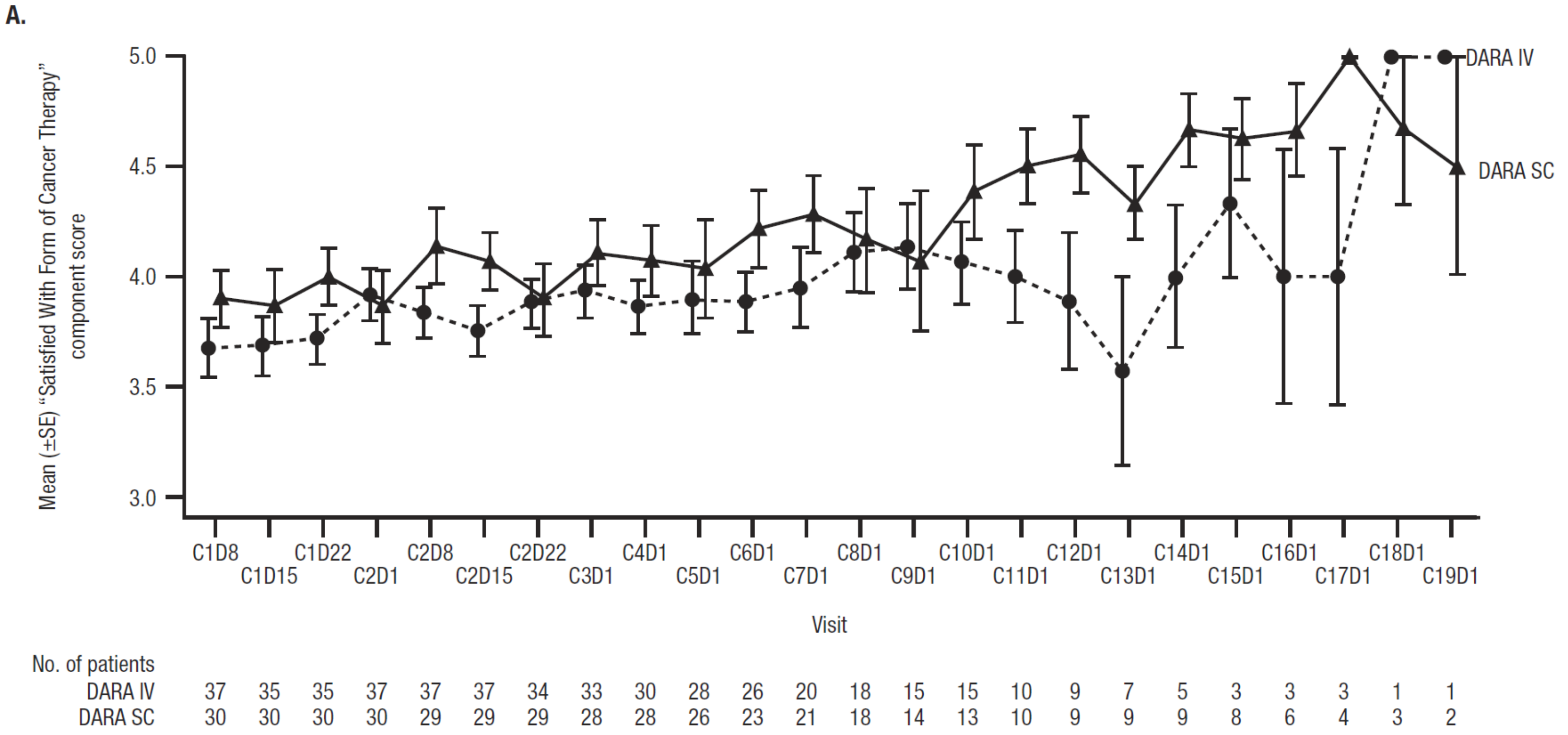

B.

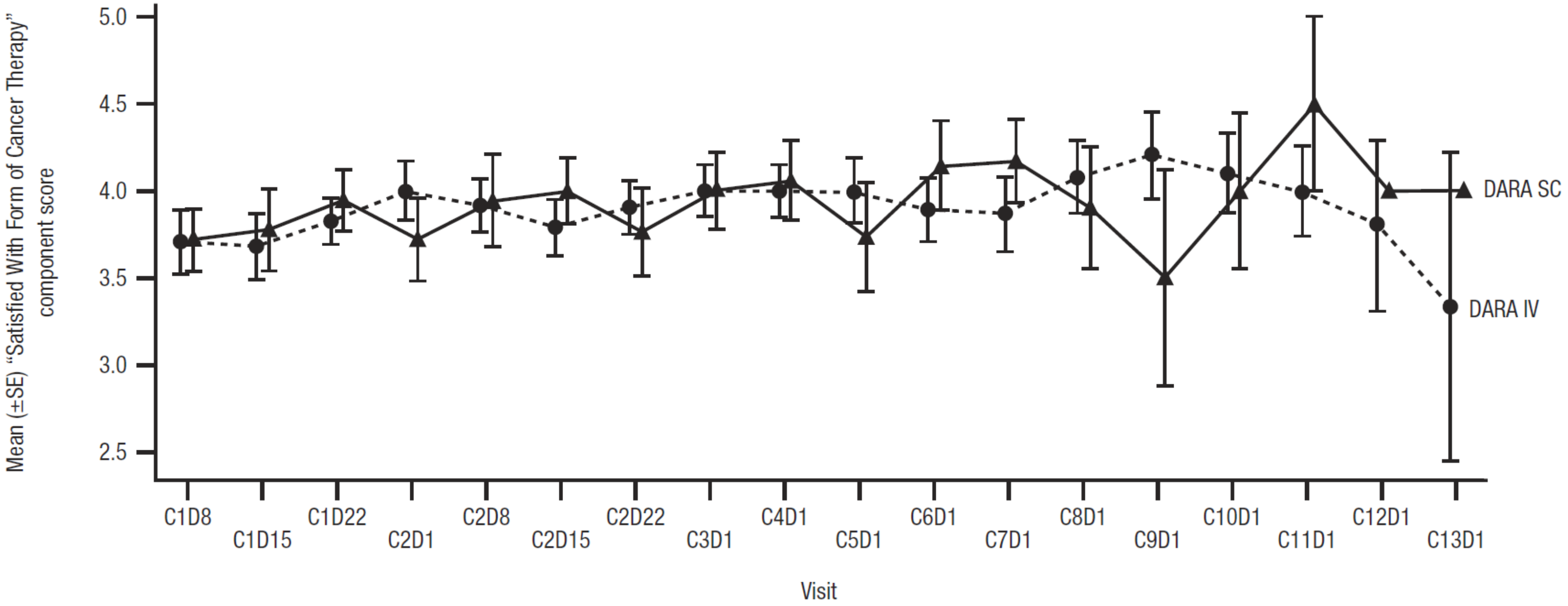

No. of patients																		
DARA IV	24	22	23	24	24	24	21	21	20	19	18	15	13	10	10	6	5	3
DARA SC	18	18	18	18	17	17	17	16	16	15	14	12	10	6	5	2	1	1

CTSQ, Cancer Therapy Satisfaction Questionnaire; SE, standard error; C, cycle; D, day; DARA, daratumumab; IV, intravenous; SC, subcutaneous.

Supplementary Fig. 5 Mean modified CTSQ scores over time for the "Taking Cancer Therapy as Difficult as Expected" component of the Satisfaction With Therapy domain in (a) Asian and (b) Japanese patients

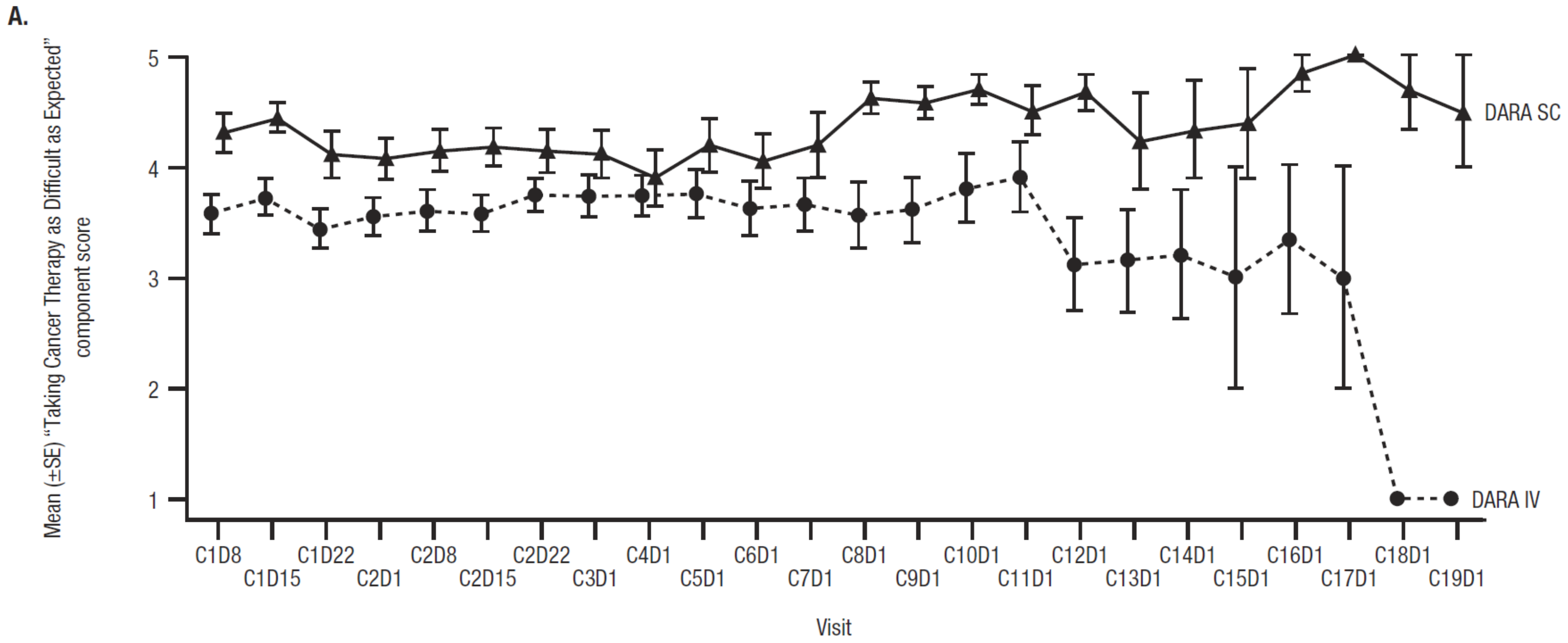

No. of patients																								
DARA IV	37	35	35	37	37	37	34	33	30	28	26	20	18	15	15	10	9	7	5	3	3	3	1	1
DARA SC	30	30	30	30	29	29	29	28	28	26	23	21	18	14	13	10	9	9	9	8	6	4	3	2

B.

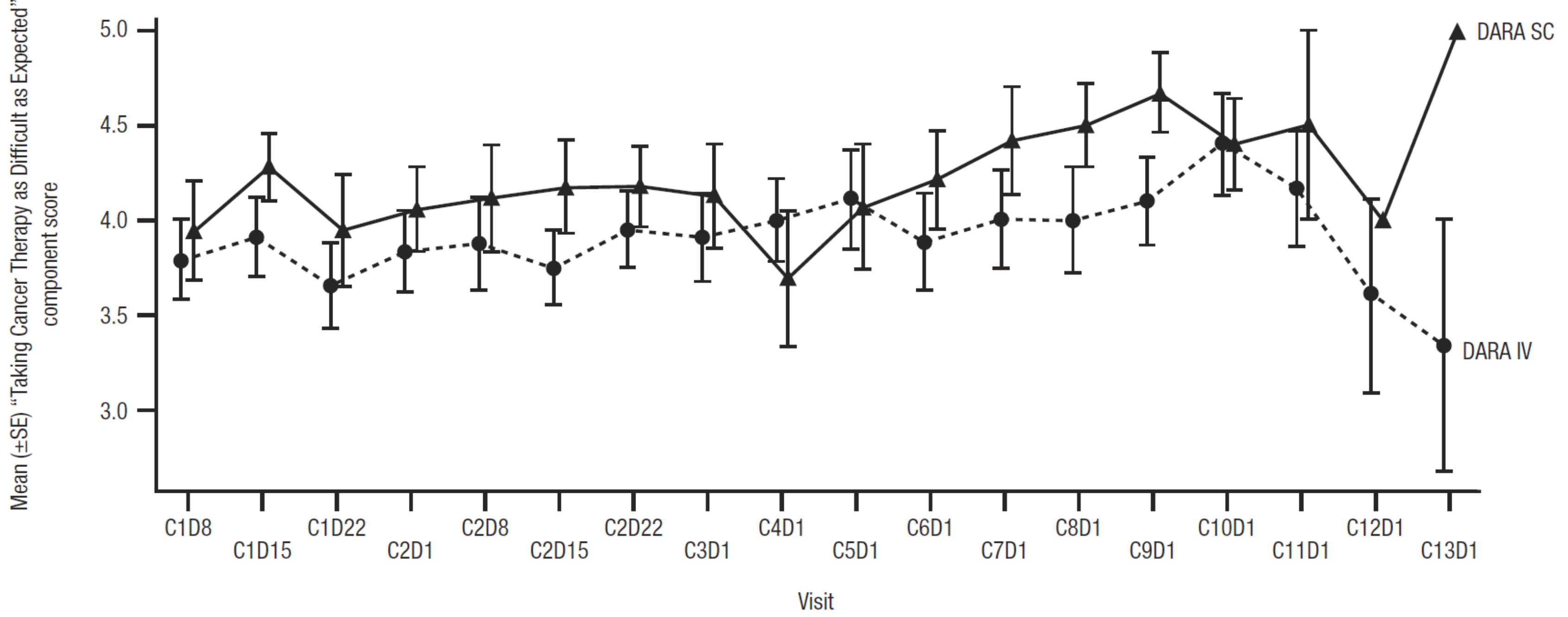

No. of patients																		
DARA IV	24	22	23	24	24	24	21	21	20	19	18	15	13	10	10	6	5	3
DARA SC	18	18	18	18	17	17	17	16	16	15	14	12	10	6	5	2	1	1

CTSQ, Cancer Therapy Satisfaction Questionnaire; SE, standard error; C, cycle; D, day; DARA, daratumumab; IV, intravenous; SC, subcutaneous.
